# The polymorphisms (rs3213801 and rs5744533) of DNA polymerase kappa gene are not related with glioma risk and prognosis: A case‐control study

**DOI:** 10.1002/cam4.2566

**Published:** 2019-10-08

**Authors:** Ying Wu, Linghui Zhou, Yujiao Deng, Na Li, Pengtao Yang, Shanshan Dong, Si Yang, Yi Zheng, Li Yao, Ming Zhang, Zhen Zhai, Zhijun Dai, Yuan Wu

**Affiliations:** ^1^ Department of Oncology The Second Affiliated Hospital of Xi'an Jiaotong University Xi'an China; ^2^ School of Life Science and Technology Xi'an Jiaotong University Xi'an China; ^3^ Department of Neurology The Second Affiliated Hospital of Xi'an Jiaotong University Xi'an China; ^4^ Department of Neurosurgery The Second Affiliated Hospital of Xi'an Jiaotong University Shannxi China; ^5^ Department of Critical Care Medicine The Second Affiliated Hospital of Xi'an Jiaotong University Shannxi China

**Keywords:** glioma, *POLK*, polymorphisms, prognosis, susceptibility

## Abstract

DNA polymerase kappa (*POLK*), one of the specialized Y family DNA polymerases, functions in translesion synthesis and is suggested to be related with cancers. Single nucleotide polymorphisms (SNPs) in specialized DNA polymerases have been demonstrated to be associated with cancer risk. To evaluate the association of two common *POLK* variants (rs3213801 C>T and rs5744533 C>T) with glioma, we conducted a case‐control study and genotyped these two *POLK* variants in 605 patients and 1300 healthy controls. The association analysis revealed no significant correlations were observed between these two *POLK* SNPs and glioma risk. However, the *POLK* rs3213801 CT genotype was found to be higher in older glioma patients (≥40) than in younger patients (*P* = .026). Compared with patients harboring the CC genotype, the frequencies of *POLK* rs5744533 CT and CT+TT genotypes were increased in patients with lower World Health Organization (WHO) grade glioma (*P* = .028, 0.044, respectively). According to Kaplan‐Meier analysis and log‐rank tests, *POLK* SNPs were not correlated with either the overall survival or progression‐free survival. Nevertheless, multivariate analysis revealed that the age (≥40) could increase the risk of death in glioma patients (*P* < .05), while gross‐total resection and temozolomide treatment were found to play protective roles in glioma prognosis (*P* < .001, respectively). Overall, our results indicated that *POLK* variants rs3213801 and rs5744533 are not associated with glioma risk and prognosis. However, these polymorphisms are likely to be associated with certain glioma characteristics, such as age and WHO grade. The age, surgery types, and chemotherapy could be independent prognostic factors in glioma. More studies are required to confirm our findings.

## Graphical abstract

In this study, we found DNA polymerase kappa variants rs3213801 and rs5744533 are not associated with glioma risk and prognosis, but they are likely to be associated with certain glioma characteristics, such as age and World Health Organization grade. Meanwhile, the age, surgery types, and chemotherapy could be independent prognostic factors in glioma.

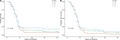

## 1 INTRODUCTION

Gliomas, which develop from glial or precursor cells, account for 75% of primary malignant primary brain tumors in adults. Gliomas include astrocytomas, oligodendrogliomas, and ependymomas.^1^ The World Health Organization (WHO) classified gliomas into four malignant grades (I‐IV) based on the extent of cell proliferation, angiogenesis, and necrosis.^2^ Surgery is the primary method for the treatment of gliomas, although alternative treatments are available, such as chemotherapy, radiotherapy, and immunotherapy. However, treatment of malignant brain tumors remains difficult with an overall survival (OS) less than 35%.^1^ Consistent with findings obtained for other types of cancer, genomic alterations are widely recognized to induce glioma initiation, in which DNA damage might play an important role.

DNA damage from different sources, such as UV light, ionizing radiation, chemical poisons and drugs, and reactive oxygen species that are byproducts of routine metabolic processes, is a key process underlying tumorigenesis.^3^ Meanwhile, DNA replication is a requisite for cancer progression. During DNA replication, DNA repair mechanisms help minimize the consequences of DNA damage through base excision repair and nucleotide excision repair.^4^ However, if the DNA damage could not be repaired, specialized DNA polymerases (*β*, *ι*, *κ*) function in translesion synthesis (TLS) by inserting a base opposite a lesion to reverse the damage.^5^ The DNA replication polymerase becomes stalled and is then replaced by the specialized polymerase at the site of DNA damage.^6^ DNA double‐strand breaks are generated in the absence of TLS, which increases genomic instability and leads to the collapse of the stalled replication forks.^4^, ^7^ DNA polymerase kappa (*POLK*), a specialized DNA polymerase and member of Y family, can synthesize DNA with higher fidelity compared to other Y‐family members.^8^, ^9^ Previous studies suggested that *POLK* is associated with some cancers, such as non‐small cell lung cancer (NSCLC) and kidney cancer and can induce genetic alterations.^10^, ^11^, ^12^

Single nucleotide polymorphisms (SNPs) are widely recognized to be associated with genetic susceptibility to cancers, and they are also demonstrated to be vital to neurologic tumors risk.^13^ Previous studies indicated that *POLK* harbors various SNPs, which are considered to be associated with various cancer risk.^14^, ^15^ At the same time, *POLK* was demonstrated to be strongly upregulated in glioma and is strongly associated with the clinical outcomes of glioma patients, indicating that *POLK* could serve as an independent prognostic marker for glioma.^16^ Nevertheless, the correlation between *POLK* polymorphisms and glioma has not been identified. Therefore, we genotyped two cancer‐risk related SNPs of *POLK* (rs3213801 C>T and rs5744533 C>T) in 650 glioma patients and 1300 controls to explore their associations with glioma risk.

## 2 METHODS

### 2.1 Study populations

This case‐control study included 605 glioma patients and 1300 controls, consecutively. No age restrictions were applied for the recruitment. All glioma patients were diagnosed at the Department of Neurosurgery of Tangdu Hospital, Fourth Military Medical University, Shaanxi Province (Xi'an, China) from September 2010 to May 2014. Patients were excluded if they had other nervous system diseases or other types of cancer. Similarly, we excluded patients who received prior treatment, such as chemotherapy and radiotherapy, prior to surgery. In the control group, 1300 cancer‐free controls without any history of underlying diseases were recruited from a group of participants who were receiving routine examinations in the outpatient department at the hospital. Self‐administered questionnaires were used to collected relevant information of all participants, including ethnicity, age, and gender, through interviewing after obtaining written informed consents. Clinical information, including the surgery record and treatment method, was obtained from the medical records, and patient condition and pathology results were updated during follow‐up sessions. For analyzing the prognosis of these glioma patients, the follow‐up was performed every month through telephone interviews, communications with patients' families, and outpatient visits.

### 2.2 SNP selection, DNA extraction, and genotyping

According to the NCBI database, *POLK* rs3213801 C>T and rs5744533 C>T respectively located in exon coding sequence and upper stream sequence of *POLK* gene (https://www.ncbi.nlm.nih.gov/snp). Previous studies proved that *POLK* rs3213801 and rs5744533 were significantly associated with breast cancer and lung cancer susceptibility.^15^, ^17^, ^18^ In the meantime, owing to high expression of *POLK* and its prognostic value in glioma, we finally selected these two cancer‐risk associated *POLK* polymorphisms, namely, rs3213801 C>T and rs5744533 C>T, to explore the associations via a case‐control study.

In this study, we collected blood samples from patients and controls in tubes containing EDTA. After centrifuging the samples, we extracted genomic DNA according to the standard phenol‐chloroform extraction procedure following previously described methods.^19^, ^20^ DNA concentration was measured using Spectrometry (DU530 UV/VIS spectrophotometer; Beckman Instruments). In the process of designing a Multiplexed SNP Mass EXTEND assay, we used Sequenom Mass ARRAY Assay Design 3.0 Software (Sequenom, Inc). Genotyping of *POLK* polymorphisms was conducted through Sequenom Mass ARRAYRS1000 according to manufacturer's instructions. The primers used in our study are listed in Table S1. Data were analyzed by Sequenom Typer 3.0 Software (Sequenom, Inc). According to the results, the response rate of 100% was observed for both glioma patients and cancer‐free controls.

### 2.3 Statistical analysis

All experimental data were analyzed via SPSS 18.0. Hardy‐Weinberg equilibrium (HWE) was tested using chi‐square test. The distributions of the genotype frequencies of the two SNPs between patients and healthy controls were evaluated through Student's *t* test or *χ* ^2^ test. Odds ratios (ORs) with their 95% confidence intervals (CIs) were computed to estimate the relationship between *POLK* polymorphisms and the clinical characteristics of patients. The effect of different genotypes of *POLK* SNPs on glioma prognosis was estimated by performing Kaplan‐Meier analysis and log‐rank tests. Univariate and multivariate Cox analysis were conducted to assess the prognostic roles of various factors in glioma patients, and hazard ratios (HRs) and 95% CIs were calculated. *P* less than .05 was recognized to be statistically significant. All statistical tests were two‐tailed.

## 3 RESULTS

### 3.1 Characteristics of study subjects

All participants included in the study were Han Chinese. The characteristics of patients and control groups are described in Table S2. The two groups showed no significant differences in age and sex (*P* = .195, .688, respectively). We considered the 382 glioma patients (63.1%) with WHO Grade I‐II under the low‐grade glioma group, whereas the 223 patients (36.9%) with WHO Grade III‐IV were classified under the high‐grade glioma group. Among these patients, a total of 416 (68.8%) patients underwent gross‐total resection (GTR) and 189 (31.2%) patients underwent subtotal resection (STR) or near total resection (NTR). Except 60 patients who did not receive any radiotherapy, a total of 545 (90.1%) patients received radiotherapy (162 patients received conformal radiotherapy and 383 patients received gamma knife). There were 250 (41.3%) patients who received chemotherapy, in which 124 patients received platinum treatment, 52 patients received temozolomide treatment, and 74 patients received nimustine treatment, whereas 355 patients did not receive any chemotherapy.

### 3.2 Correlations between *POLK* SNPs and glioma risk

The genotype distributions and allele frequencies of the *POLK* rs3213801 and rs5744533 polymorphisms are summarized in Table 1. The genotypic frequencies for the *POLK* polymorphisms (rs3213801 and rs5744533) in controls conformed to the HWE (*P* = .50, .42, respectively), which indicated that the controls could represent the general population. Results revealed no significant associations between any genotype of these two SNPs and glioma risk.

**Table 1:** Genotype frequencies of *POLK* polymorphisms in cases and controls

Model	Genotype	Control (n, %)	Case (n, %)	OR (95% CI)	*P*‐value^a^
rs3213801	HWE: *P* = .50				
Co‐dominant	CC	589 (45.3)	284 (47.0)	Ref.	
Heterozygote	CT	580 (44.6)	253 (41.8)	0.90 (0.74‐1.11)	.34
Homozygote	TT	131 (10.1)	68 (11.2)	1.08 (0.78‐1.49)	.66
Dominant	CC	589 (45.3)	284 (47.0)	Ref.	
	CT+TT	711 (54.7)	321 (53.0)	0.94 (0.77‐1.14)	.51
Recessive	CC+CT	1169 (89.9)	537 (88.8)	Ref.	
	TT	131 (10.1)	68 (11.2)	1.13 (0.83‐1.54)	.44
Overdominant	CC+TT	720 (55.4)	352 (58.2)	Ref.	
	CT	580 (44.6)	253 (41.8)	0.89 (0.73‐1.08)	.25
Allele	C	1758 (67.6)	821 (67.9)	Ref.	
	T	842 (32.4)	389 (32.1)	0.99 (0.86‐1.15)	.89
rs5744533	HWE: *P* = .42				
Co‐dominant	CC	588 (45.2)	285 (47.1)	Ref.	
Heterozygote	CT	582 (44.8)	251 (41.5)	0.89 (0.73‐1.09)	.26
Homozygote	TT	130 (10.0)	69 (11.4)	1.10 (0.79‐1.52)	.58
Dominant	CC	588 (45.2)	285 (47.1)	Ref.	
	CT+TT	712 (54.8)	320 (52.9)	0.93 (0.76‐1.13)	.44
Recessive	CC+CT	1170 (90.0)	536 (88.6)	Ref.	
	TT	130 (10.0)	69 (11.4)	1.16 (0.85‐1.58)	.35
Overdominant	CC+TT	718 (55.2)	354 (58.5)	Ref.	
	CT	582 (44.8)	251 (41.5)	0.88 (0.72‐1.06)	.18
Allele	C	1758 (67.6)	821 (67.9)	Ref.	
	T	842 (32.4)	389 (32.1)	0.99 (0.86‐1.15)	.89

Abbreviations: CI, confidence interval; HWE, Hardy‐Weinberg equilibrium; OR, odds ratio; *POLK*, DNA polymerase kappa; Ref., reference.

a Univariate logistic regression analysis for the distributions of genotype and allele frequencies. Adjusted for age and sex.

### 3.3 Associations between *POLK* polymorphisms and clinical features in glioma patients

We explored the relations between *POLK* SNPs and clinical parameters in glioma cases, such as age, gender, and WHO grade. According to the results, the CT genotype frequency of *POLK* rs3213801 was higher in patients aged ≥40 (CT vs CC: OR: 1.48, 95% CI: 1.05‐2.09, *P* = .026) (Table 2). Meanwhile, compared with patients harboring the CC genotype, the CT and CT+TT genotypes of *POLK* rs5744533 were increased in the patients with lower WHO grade glioma (CT vs CC: OR = 0.67, 95%CI: 0.47‐0.96, *P* = .028; CT+TT vs CC: OR = 0.71, 95% CI:0.51‐0.99, *P* = .044) (Table 3).

**Table 2:** Associations between the *POLK* rs3213801 polymorphisms and clinical characteristics of glioma patients

Characteristics	Genotype distributions			
	CC	CT	TT	CT+TT
Age				
<40/≥40	136/148	97/156	34/34	131/190
OR (95% CI)	Ref.	1.48 (1.05‐2.09)	0.92 (0.54‐1.56)	1.33 (0.97‐1.84)
*P*‐value^a^		**.026** ^*^	.754	.08
Sex				
Male/female	163/121	126/127	46/22	172/149
OR (95% CI)	Ref.	1.36 (0.97‐1.91)	0.64 (0.36‐1.12)	1.17 (0.85‐1.61)
*P*‐value^a^		.0784	.124	.347
WHO grade				
I + II/III + IV	188/96	151/102	43/25	194/127
OR (95% CI)	Ref.	1.32 (0.93‐1.88)	1.14 (0.65‐1.96)	1.28 (0.92‐1.79)
*P*‐value^a^		.119	.644	.143

Abbreviations: CI, confidence interval; OR, odds ratio; *POLK*, DNA polymerase kappa; Ref., reference.

a Univariate logistic regression analysis for the distributions of genotype frequencies.

* *P*＜0.05

**Table 3:** Associations between the *POLK* rs5744533 polymorphisms and clinical characteristics of glioma patients

Characteristics	Genotype distributions			
	CC	CT	TT	CT+TT
Age				
<40/≥40	119/166	114/137	34/35	148/172
OR (95% CI)	1.00 (Ref.)	0.86(0.61‐1.21)	0.74(0.43‐1.25)	0.83(0.60‐1.15)
*P*‐value^a^		0.393	0.258	0.266
Sex				
Male/female	163/122	126/125	46/23	172/148
OR (95% CI)	1.00 (Ref.)	1.33 (0.94‐1.87)	0.67 (0.38‐1.15)	1.15 (0.83‐1.59)
*P*‐value^a^		0.105	0.152	0.395
WHO grade				
I + II/III + IV	168/117	171/80	43/26	214/106
OR (95% CI)	1.00 (Ref.)	0.67 (0.47‐0.96)	0.87 (0.51‐1.49)	0.71 (0.51‐0.99)
*P*‐value^a^		**0.028** ^*^	0.609	**0.044** ^*^

Abbreviations: CI, confidence interval; OR, odds ratio; *POLK*, DNA polymerase kappa; Ref., reference.

a Univariate logistic regression analysis for the distributions of genotype frequencies.

* *P*＜0.05

### 3.4 Prognostic roles of multiple factors in glioma patients

Clinical factors including age, sex, surgery types, chemotherapy, and radiotherapy were taken into accounted in univariate analysis. We found the age, surgery types, and chemotherapy were significant factors of glioma prognosis. In multivariate analysis, we determined the patients aged ≥40 had worse OS and progression‐free survival (PFS) than the younger patients (<40) (OS: HR: 1.20, 95% CI: 1.01‐1.43, *P* = .04; PFS: HR: 1.19, 95% CI: 1.01‐1.42, *P* = .042). The patients who underwent GTR had better prognosis compared with patients who received STR or NTR (OS: HR: 0.61, 95% CI: 0.50‐0.74, *P* < .001; PFS: HR: 0.59, 95% CI: 0.49‐0.72, *P* < .001). Compared with the patients who did not receive any chemotherapy, the patients who received temozolomide had more favorable OS and PFS (OS: HR: 0.31, 95% CI: 0.21‐0.47, *P* < .001; PFS: HR: 0.34, 95% CI: 0.23‐0.50, *P* < .001). Meanwhile, similar results were observed in patients who received nimustine treatment (OS: HR: 0.65, 95%CI: 0.49‐0.86, *P* = .003; PFS: HR: 0.75, 95% CI: 0.56‐0.99, *P* = .045). All the prognostic information is shown in Table S3 and S4.

**Figure 1:**
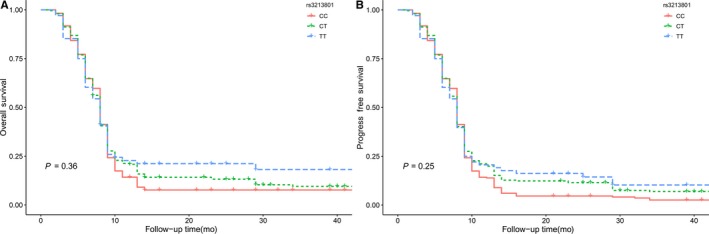
A, Association between *POLK* rs3213801 polymorphisms and patients' OS in glioma; (B) the association between *POLK* rs3213801 polymorphisms and patients' PFS in glioma. Abbreviations: *POLK*: DNA polymerase kappa; OS, overall survival; PFS, progression‐free survival

**Figure 2:**
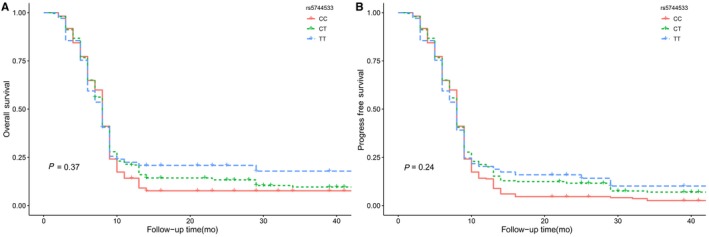
A, Association between *POLK* rs5744533 polymorphisms and patients' OS in glioma; (B) the association between *POLK* rs5744533 polymorphisms and patients' PFS in glioma. Abbreviations: *POLK*: DNA polymerase kappa; OS: overall survival; PFS: progression‐free survival

According to the Kaplan‐Meier analysis and log‐rank tests, we identified no significant associations between *POLK* rs3213801 polymorphism and prognosis of glioma patients based on OS and PFS (OS: *P* = .36; PFS: *P* = .25) (Figure 1). Similar results were obtained in glioma patients with *POLK* rs5744533 polymorphism (OS: *P* = .37; PFS: *P* = .24) (Figure 2).

## 4 DISCUSSION

As a member of specialized DNA polymerases, *POLK* participates in the process of TLS, a DNA damage tolerance mechanism. Unrepaired DNA lesions generated during DNA damage would block DNA replication, which could lead to mutations and cell death.^21^ DNA replication could be continued and the noncanonical DNA structure could be bypassed with the specialized DNA polymerases, of which each could function with different replication fidelities in humans.^22^, ^23^, ^24^ SNPs in genes encoding these specialized DNA polymerases were found to be associated with tumorigenesis and development. A meta‐analysis demonstrated that the SNP rs9333555 of DNA polymerase eta (*POLH*) gene is related with higher melanoma risk.^25^ Polymorphisms (rs8305 and rs3218786) in DNA polymerase iota (*POLI*) gene have been previously reported to be correlated with lung cancer and prostate cancer.^26^, ^27^

The associations between the alterations in *POLK* and cancers, such as lung cancer and prostate cancer, have been confirmed in multiple studies.^18^, ^28^ In our previous study, we demonstrated the relationship between *POLK* polymorphisms and breast cancer.^17^ In present study, we evaluated the value of *POLK* variants in glioma by analyzing two (rs5744533 and rs3213801) *POLK* SNPs from 605 glioma cases and 1300 controls. The results showed no significant associations in any genotype models and frequencies of the SNPs between cases and controls in *POLK* gene and glioma risk. However, we found that the frequency of *POLK* rs3213801 CT genotype was higher in patients aged ≥40 than in patients aged <40. Additionally, compared to patients harboring the CC genotype, the CT and CT+TT genotypes of *POLK* rs5744533 were increased in the patients with lower WHO grade glioma, indicating that *POLK* polymorphisms might exert protective functions in the development of glioma. Furthermore, the prognostic value of various factors in glioma was investigated. According to the results of univariate and multivariate analysis, age, surgery types, and chemotherapy were found to be independent risk factors in glioma. Older glioma patients (≥40) had higher risk of death on OS and PFS compared to patients aged <40. On the contrary, better prognosis was found in the patients who underwent the GTR or received chemotherapy including temozolomide and nimustine treatment. Similarly, previous studies demonstrated that extend of resection and chemotherapy played protective roles in glioma.^29^, ^30^, ^31^ As for the prognostic value of *POLK* SNPs in glioma, a similar study by Shao et al. showed no significant associations between *POLK* SNPs (rs3213801 and rs5744533) and patient prognosis in NSCLC.^18^ Consistent with the above findings, we failed to identify statistically significant differences between genotypes of *POLK* SNPs and survival of glioma patients.

Our results could be explained by the following limitations. First, the sample size was insufficient, and the two groups were unbalanced. A larger sample size and additional glioma cases should be incorporated to increase the statistical significance. Second, considering the lack of data availability, other factors, such as high‐dose radiation exposure, lifestyle, heredity history, and tumor size, were not considered. Third, all the patients were recruited from the northwest of China, which could have contributed to selection bias. However, although our findings did not support the correlations between *POLK* polymorphisms and glioma in Han Chinese population, the possibility that the *POLK* polymorphisms confer genetic susceptibility to glioma in other populations cannot be excluded.

To sum up, the results of this case‐control study showed no statistically significant associations between *POLK* SNPs (rs3213801 and rs5744533) and glioma, which indicated that *POLK* might not contribute to the glioma susceptibility in the Han population of northwest of China. In addition, we identified no significant associations between *POLK* polymorphisms and the prognosis of glioma patients. Despite the negative results, further studies that investigate *POLK* SNPs in glioma would be worthwhile. Herein, the two *POLK* SNPs could be associated with older patients or patients who have lower glioma grade. Meanwhile, the age, surgery types and chemotherapy could be independent prognostic factors in glioma. Further studies on *POLK* polymorphisms and multi‐central studies are expected to provide more insights into the relationships between *POLK* SNPs and glioma risk.

## CONFLICTS OF INTERESTS

None declared.

## AUTHORS' CONTRIBUTIONS

YW, LH Z, and YJ D designed the study and prepared the manuscript. Y W, N L, SS D, and Z Z took responsibility for preparation of data. S Y, Y Z, and L Y analyzed the data. M Z and PT Y contributed materials and analysis tools. ZJ D and Y W were involved in manuscript revision. All authors read and approved the final manuscript.

## ETHICAL APPROVAL

The protocol of this study was approved by the Ethics Committee of the Institutional Review Board of Xi'an Jiaotong University. All participants had read and signed the written informed consents in this study.
