# Supplementary material for: The polymorphisms (rs3213801 and rs5744533) of DNA polymerase kappa gene are not related with glioma risk and prognosis: A case‐control study
Source: Cancer Med. 2019 Oct 8;8(17):7446–53. doi: 10.1002/cam4.2566 (PMC6885875; doi:10.1002/cam4.2566)
Supplement: Supplementary file 1 [file CAM4-8-7446-s001.docx]

**Supplementary materials:**

**Table S1.** Primers used for this study.

| **SNP_ID** | **1st-PCRP** | **2nd-PCRP** | **UEP_SEQ** |
| --- | --- | --- | --- |
| **rs3213801** | ACGTTGGATGTACAGTGGCCATCAATACTC | ACGTTGGATGGAACATCAGCCCAGGAAGTG | CTCCCAAAACAACACTGACAGC |
| **rs5744533** | ACGTTGGATGTGCAGGTGGATGATCTCTTG | ACGTTGGATGGAGAAGTCAAAAAGGCTACG | GGCTACGTGAATCTTCTTA |

**Table S2.** The characteristics of glioma cases and cancer-free controls.

| **Characteristics** | **Cases** | **Control** | ***P-*value**^†^ |
| --- | --- | --- | --- |
| Number | 605 | 1300 |  |
| **Age** (mean ± SD) | 40.71±18.28 | 41.68±13.54 | 0.195 |
| ＜40 years | 267 | 561 |  |
| ≥40 years | 338 | 739 | 0.688 |
| **Sex** | | | |
| Male | 335 | 700 |  |
| female | 270 | 600 | 0.534 |
| **WHO Grade** | | | |
| I-II | 382 |  |  |
| III-IV | 223 |  |  |
| **Surgery** |  |  |  |
| STR & NTR | 189 |  |  |
| GTR | 416 |  |  |
| **Radiotherapy** |  |  |  |
| No | 60 |  |  |
| Yes | 545 |  |  |
| **Chemotherapy** |  |  |  |
| No | 355 |  |  |
| Yes | 250 |  |  |

^†^ T-test or two-sided χ^2^-test.

**Abbreviations:** STR: subtotal resection; NTR: near total resection; GTR: gross total resection.

**Table S3.** Univariate and multivariate analysis of the associations of various factors and glioma patients’ OS

| **Characteristics** | **Patients, n** | **Events, n** | **Rate, %** | **Univariate Analysis** | | **Multivariable Analysis** | |
| --- | --- | --- | --- | --- | --- | --- | --- |
|  |  |  |  | **HR (95% CI)** | ***P*-Value**^†^ | **HR (95% CI)** | ***P*-Value**^†^ |
| **Age** |  |  |  |  |  |  |  |
| <40 years | 267 | 229 | 85.77 | Ref. | Ref. | Ref. | Ref. |
| >=40 years | 338 | 310 | 91.72 | **1.20(1.01-1.42)** | **0.039^*^** | **1.21(1.02-1.44)** | **0.029^*^** |
| **Sex** |  |  |  |  |  |  |  |
| male | 335 | 297 | 88.66 | Ref. | Ref. |  |  |
| female | 270 | 242 | 89.63 | 1.08(0.91-1.28) | 0.355 |  |  |
| **WHO Grade** |  |  |  |  |  |  |  |
| I-II | 382 | 336 | 87.96 | Ref. | Ref. |  |  |
| III-IV | 223 | 206 | 92.38 | 1.18(0.98- 1.40) | 0.063 |  |  |
| **Surgery** |  |  |  |  |  |  |  |
| STR & NTR | 189 | 186 | 98.41 | Ref. | Ref. | Ref. | Ref. |
| GTR | 416 | 353 | 84.86 | **0.59(0.49-0.71)** | **<0.001^***^** | **0.62(0.51-0.75)** | **<0.001^***^** |
| **Chemotherapy** |  |  |  |  |  |  |  |
| No | 355 | 333 | 93.80 | Ref | Ref | Ref | Ref |
| Platinum | 124 | 112 | 90.32 | 0.84(0.68-1.04) | 0.116 | 0.82(0.66-1.02) | 0.072 |
| Temozolomide | 52 | 30 | 57.69 | **0.32(0.22-0.48)** | **<0.001^***^** | **0.36(0.24-0.52)** | **<0.001^***^** |
| Nimustine | 74 | 64 | 86.49 | **0.645(0.49-0.85)** | **0.001^**^** | **0.74(0.56-0.97)** | **0.030^*^** |
| **Radiotherapy** |  |  |  |  |  |  |  |
| No | 60 | 49 | 81.67 | Ref. | Ref. |  |  |
| Conformal radiotherapy | 162 | 133 | 82.10 | 1.08 (0.77-1.50) | 0.622 |  |  |
| Gamma knife | 383 | 357 | 93.21 | 1.17 (0.86-1.58) | 0.303 |  |  |
| **rs3213801** |  |  |  |  |  |  |  |
| CC | 284 | 262 | 92.25 | Ref. | Ref. |  |  |
| CT | 253 | 223 | 88.14 | 0.91(0.76-1.09) | 0.32 |  |  |
| TT | 68 | 54 | 79.41 | 0.82(0.61-1.11) | 0.196 |  |  |
| **rs5744533** |  |  |  |  |  |  |  |
| CC | 285 | 263 | 92.28 | Ref. | Ref. |  |  |
| CT | 251 | 221 | 88.05 | 0.91(0.76-1.09) | 0.301 |  |  |
| TT | 69 | 55 | 79.71 | 0.84(0.62-1.12) | 0.226 |  |  |

^†^ Cox’s proportional hazard regression analysis for univariate and multivariate analysis. **P*＜0.05 ***P*＜0.01 ****P*＜0.001

**Abbreviations:** OS: overall survival; HR: hazard ratio; CI: confidence interval; STR: subtotal resection; NTR: near total resection; GTR: gross total resection; Ref.: reference.

**Table S4.** Univariate and Multivariate analysis of the associations of various factors and glioma patients’ PFS

| **Characteristics** | **Patients, n** | **Events, n** | **Rate, %** | **Univariate Analysis** | | **Multivariable Analysis** | |
| --- | --- | --- | --- | --- | --- | --- | --- |
|  |  |  |  | **HR (95% CI)** | ***P*-Value**^†^ | **HR (95% CI)** | ***P*-Value**^†^ |
| **Age** |  |  |  |  |  |  |  |
| <40 years | 267 | 239 | 89.51 | Ref. | Ref. | Ref. | Ref. |
| >=40 years | 338 | 324 | 95.86 | 1.19(1.00-1.40) | 0.047 | **1.21 (1.02-1.43)** | **0.030^*^** |
| **Sex** |  |  |  |  |  |  |  |
| male | 335 | 310 | 92.54 | Ref. | Ref. |  |  |
| female | 270 | 253 | 93.70 | 1.1(0.93-1.30) | 0.263 |  |  |
| **WHO Grade** |  |  |  |  |  |  |  |
| I-II | 382 | 353 | 92.41 | Ref. | Ref. |  |  |
| III-IV | 223 | 210 | 94.17 | 1.15(0.97-1.36) | 0.116 |  |  |
| **Surgery** |  |  |  |  |  |  |  |
| STR & NTR | 189 | 183 | 96.83 | Ref. | Ref. | Ref. | Ref. |
| GTR | 416 | 380 | 91.35 | **0.58(0.48-0.69)** | **<0.001^***^** | **0.61(0.51-0.74)** | **<0.001^***^** |
| **Chemotherapy** |  |  |  |  |  |  |  |
| No | 355 | 351 | 98.87 | Ref. | Ref. | Ref. | Ref. |
| Platinum | 124 | 116 | 93.55 | 0.99(0.80-1.22) | 0.916 | 0.98(0.79-1.21) | 0.85 |
| Temozolomide | 52 | 32 | 61.54 | **0.35(0.24-0.50)** | **<0.001^***^** | **0.38(0.26-0.55)** | **<0.001^***^** |
| Nimustine | 74 | 64 | 86.49 | **0.73(0.56-0.96)** | **0.022^*^** | 0.83(0.63-1.10) | 0.189 |
| **Radiotherapy** |  |  |  |  |  |  |  |
| No | 60 | 55 | 91.67 | Ref. | Ref. |  |  |
| Conformal radiotherapy | 162 | 137 | 84.57 | 1.13(0.83-1.56) | 0.436 |  |  |
| Gamma knife | 383 | 371 | 96.87 | 1.21(0.91-1.60) | 0.199 |  |  |
| **rs3213801** |  |  |  |  |  |  |  |
| CC | 284 | 272 | 95.77 | Ref. | Ref. |  |  |
| CT | 253 | 231 | 91.30 | 0.87(0.73-1.04) | 0.123 |  |  |
| TT | 68 | 60 | 88.24 | 0.84(0.63-1.11) | 0.217 |  |  |
| **rs5744533** |  |  |  |  |  |  |  |
| CC | 285 | 273 | 95.79 | Ref. | Ref. |  |  |
| CT | 251 | 229 | 91.24 | 0.87(0.73-1.03) | 0.11 |  |  |
| TT | 69 | 61 | 88.41 | 0.85(0.64-1.12) | 0.242 |  |  |

^†^ Cox’s proportional hazard regression analysis for univariate and multivariate analysis. **P*＜0.05 ****P*＜0.001

**Abbreviations:** PFS: progression-free survival; HR: hazard ratio; CI: confidence interval; STR: subtotal resection; NTR: near total resection; GTR: gross total resection; Ref.: reference.
